# Multiscale Approach to the Determination of the Photoactive Yellow Protein Signaling State Ensemble

**DOI:** 10.1371/journal.pcbi.1003797

**Published:** 2014-10-30

**Authors:** Mary A. Rohrdanz, Wenwei Zheng, Bradley Lambeth, Jocelyne Vreede, Cecilia Clementi

**Affiliations:** 1 Center for Theoretical Biological Physics, Rice University, Houston, Texas, United States of America; 2 Chemistry Department, Rice University, Houston, Texas, United States of America; 3 van't Hoff Institute for Molecular Sciences, University of Amsterdam, Amsterdam, The Netherlands; University of Houston, United States of America

## Abstract

The nature of the optical cycle of photoactive yellow protein (PYP) makes its elucidation challenging for both experiment and theory. The long transition times render conventional simulation methods ineffective, and yet the short signaling-state lifetime makes experimental data difficult to obtain and interpret. Here, through an innovative combination of computational methods, a prediction and analysis of the biological signaling state of PYP is presented. Coarse-grained modeling and locally scaled diffusion map are first used to obtain a rough bird's-eye view of the free energy landscape of photo-activated PYP. Then all-atom reconstruction, followed by an enhanced sampling scheme; diffusion map-directed-molecular dynamics are used to focus in on the signaling-state region of configuration space and obtain an ensemble of signaling state structures. To the best of our knowledge, this is the first time an all-atom reconstruction from a coarse grained model has been performed in a relatively unexplored region of molecular configuration space. We compare our signaling state prediction with previous computational and more recent experimental results, and the comparison is favorable, which validates the method presented. This approach provides additional insight to understand the PYP photo cycle, and can be applied to other systems for which more direct methods are impractical.

## Author Summary

Many protein systems of biological interest undergo dynamical changes on a time scale too long to be modeled using standard computational methods. One example is photoactive yellow protein (PYP), found in several bacterial species. Blue light, potentially harmful for DNA, triggers several structural changes in PYP, eventually resulting in a conformation that changes the swimming behavior of bacteria. This conformation is difficult to investigate, as it is too short lived. In addition, understanding this “signaling state” is computationally difficult because of the long timescale of the transition. We overcome this by constructing a coarse-grained model to rapidly induce transitions to the signaling state. We then reconstruct and further sample the all-atom configurations from these coarse-grained representations. Our results are consistent with all available experimental and computational evidence.

## Introduction

Photoactive yellow protein (PYP) was first discovered in the negative phototaxis of the bacterium *Halorhodospira halophilia* [1], [2]. Upon absorption of a blue photon in solution, the protein undergoes a large structural rearrangement to form a signaling state. This conformation of the protein is thought to trigger the bacterium to avoid potentially harmful blue light. The explicit details of the transduction from blue light to physical motion of the bacterium are unknown. In addition to its biological interest, PYP is a popular model signaling protein due to its small size. As such, PYP has found use as a prototype biosensor [3], and in procedures for quantifying protein expression [4]. In addition PYP and its circularly permuted variants have been used as a photo switch [5]–[7].

Upon absorption of a blue photon, the para-coumaric acid (pCA) chromophore, which is covalently bound to CYS69, undergoes a *trans*- to *cis*- isomerization (on a picosecond timescale [8]). The isomerization makes favorable a proton transfer from GLU46 to the chromophore, and disrupts the hydrogen bonding network within the core of the protein. In solution this results in a partial unfolding, which passes through several metastable intermediates on microsecond timescales, and finally to the signaling state pB on a millisecond timescale. Several hundred milliseconds later, the initial state, the pG state, of the protein is recovered, with the pCA returned to its unprotonated *trans* configuration (see Kim, et al. for recent dynamical experiments [9]). These processes occur at a challenging set of timescales: the partial unfolding of the protein is too long to analyze with conventional molecular dynamics, and yet the lifetime of the pB signaling state is too brief to extract a detailed structure from direct experiment. In addition, the details of the PYP photo cycle are strongly environmentally dependent. In solution the photo-activated state is structurally quite different from the dark state, somewhat extended and with more disordered regions [10], while in crystallographic experiments the photo-induced changes are localized near the chromophore [11]. In solution, the kinetics are related to proton absorption and release by chromophore [12], and therefore factors such as pH [13]–[15], and salt concentration [16], [17] affect the dynamical structural changes.

Despite these difficulties, a large amount of experimental and computational work has been performed relating to the solution-phase PYP photo cycle. The structure of the dark pG state has been determined by NMR spectroscopy [18], and the resulting structures are in the Protein Data Bank (PDB) [19], with PDB ID: 3PHY. NMR experiments have characterized the amount of disorder in pB state [10]. Fourier transform infrared spectroscopy experiments have analyzed water motion during the photo cycle [20], and the initial structural changes after photon absorption [21]. Denaturation experiments on WT-PYP and mutants with the first 25 and 27 residues removed were performed to determine the effect of these first residues on the stability of the various states of the protein [22], along with small-angle X-ray scattering experiments on similarly N-terminally truncated versions of PYP [23], [24]. Circular dichroism spectroscopy experiments show an alteration and decrease in tertiary and secondary structure upon formation of the pB state [25], [26].

A feature of PYP that is not completely understood is the fact that the aforementioned N-terminally truncated mutants, while undergoing a similar photo cycle, have a less stable pG state and a much longer-lived signaling state. Indeed, some of these shortened PYP variants unfold at room temperature without any initial photo-activation [22], a feature not seen in WT-PYP. The most well-characterized mutant has the first 25 residues removed, termed Δ25, and has a signaling state lifetime ∼100 times longer than WT-PYP [22]. For this mutant solution-phase NMR structural measurements have been performed and added to the PDB, PDB ID: 1XFQ [27].

More recently, a combination of experimental techniques have been able to provide more detailed information on the solution-phase structure of the signaling state in WT-PYP. In these experiments data from double electron electron resonance spectroscopy (DEER), NMR, and time-resolved pump-probe X-ray solution scattering (TR-SAXS/WAXS) are analyzed together to yield higher-resolution structures (PDB ID: 2KX6 [28]). These results are essentially in agreement with the aforementioned NMR measurements on Δ25; both sets of experiments show pB structures that conserve much of the central *β* sheet present in the pG state and the *α* helix in residues 76–86, but have the *α* helix in residues 43–51 (the *α*_3_ helix) unformed. These WT-PYP experiments include structural information on the N-terminus region, which is shown to extend away from the globular part of the protein in the signaling configurations. In addition, pump-probe X-ray solution scattering experiments have provided kinetic and structural information on the various photo cycle intermediates [9]. As will be shown below, the structural features found here are in accord with those of reference [28].

Computational studies have also been performed on this system. Molecular mechanics combined with quantum chemical calculations have provided information on the effect of the protein environment on the chromophore, and shows that after pCA isomerization, the initiating step for disruption of the hydrogen bonding network within the chromophore binding pocket is the proton transfer from GLU46 to pCA [29], [30]. Additional quantum mechanics/molecular mechanics (QM/MM) simulations further elucidated the initial events that occur after photon absorption, showing the energetic details of the pCA isomerization and changes in the hydrogen bonding network within the chromophore binding pocket [31].

The timescale for the pG 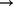 pB transition is too long to be studied by conventional all-atom molecular dynamics (MD). However parallel tempering calculations have provided a prediction of the pB configuration [32], as well as a comparison of the dynamics of Δ25 and WT-PYP [33]. Due to the very long timescales and high free energy barriers in the PYP landscape, even these calculations were not fully converged. In addition, transition path sampling combined with maximum likelihood analysis has determined good reaction coordinates for the transition from the pG to the pB ensemble [34]; we use these coordinates in part of our analysis below. These results are in general agreement with the above-mentioned experiments; however uncertainties and discrepancies remain, mostly concerning the role of the first 25 N-terminal residues. The results we present here are in agreement with previous calculations, as discussed below. In addition, they compare favorably with more recent detailed experimental results [28], which were published subsequent to the relevant computational results [32]. We predict this overall strategy to be useful in other situations with long timescales and unknown metastable states. In the following subsection we explain the general technique, discussing the various steps involved. The Results and Discussion section presents an analysis of the approach applied to PYP, followed by the Conclusions. The Materials and Methods section provides the technical aspects of the calculations.

### The overall approach

The view emerging from previous experiments and calculations is that the pG free energy basin is stabilized by the hydrogen bonding network within the chromophore binding pocket, and disrupting these bonds creates population in an alternative minimum: the signaling state. Within the energy landscape theory perspective [35], PYP can be seen as a somewhat frustrated energy landscape with two main basins. Alteration of the isomerization state of the chromophore and consequent disruption of the hydrogen bonding network shifts the relative stability of the basins from pG (for *trans* pCA) to pB (for *cis* pCA). The main coarse features of this landscape should be captured with an appropriate coarse-grained potential, and we use such a potential below to initiate the search for a signaling state ensemble in the pB basin.

Coarse-grained modeling has become a popular technique [36], [37] due to the complexity and long timescales involved in biological systems. Such methods speed computational simulation times by combining several atoms into a single bead. The method we use here was first presented in reference [38], and is hereafter called the DMC method (after Das, Matysiak, and Clementi) [39], [40]. The DMC model represents each amino acid type as a different ‘color’ bead, centered on the backbone 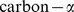, and with color-specific interactions between beads. The fact that alteration of a single PYP residue produces such a large change in the global free energy landscape, namely the population of an alternative minimum, suggests that non-native interactions are an important feature for this system. Therefore, we expect that structure-based models in which only native contacts are energetically favorable [41] are unable to capture the essential features of this system. Indeed application of such models to PYP did not produce any pB-like minimum in the free energy, only a pG-like and a globally unfolded minimum. Multiple-basin structure-based models have been developed [42]; however these require the structures in each minimum to be known a priori. The only information the DMC model explicitly requires is the pG state configuration (taken from the PDB) and the knowledge that the chromophore is exposed to the solvent in the pB signaling-state configuration. The DMC model can be considered a “first-order” correction to structure-based models, taking into account non-native interactions. Indeed, as discussed below, the DMC model produces a free energy landscape with a partially unfolded minimum in between the folded (pG) and unfolded states. Of course the DMC model of PYP does not capture the fine details of the PYP free energy landscape. Rather the model can be considered a starting point to select candidate signaling-state configurations for further analysis and exploration.

In order to find such candidate structures, the free energy landscape of the DMC PYP system is analyzed with the locally scaled diffusion map (LSDMap) [43]. LSDMap is a dimensionality reduction technique that extracts collective variables directly from simulation trajectory data, without the need for other input information such as reaction paths, intuitive coordinates, etc–which is why the technique is particularly useful for this system. The method approximates a numerical solution for the eigenfunctions of the Fokker-Planck operator, and the resulting diffusion coordinates (DCs) represent collective motions that correspond to barrier crossing processes in the system. This method was first tested on alanine dipeptide and a DMC model of src-homology 3 domain (SH3) [43], and has been applied to understand polymer reversal inside a nanopore [44], the folding pathways of a 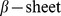 miniprotein [45], and the interaction of anthramycin and DNA [46].

From this analysis of the DMC free energy landscape, we ‘zoom in’ on the region likely to contain potential pB-like structures using an all-atom reconstruction technique: the Reconstruction Algorithm for Coarse-Grained Structures (RACOGS) [47]. There are many algorithms for reconstruction of protein side chains (e.g. [48]–[51]). The RACOGS method includes a side-chain minimization step that allows the side-chains to move continuously in space, rather than only changing between different rotamers in a library. Since rotamer libraries are typically built from datasets of native or near-native structures, this additional step makes RACOGS less likely to be biased toward native-like side-chain placements. Such a feature is important in reconstructing the non-native pB state of PYP.

The all-atom free energy landscape in the pB region of configuration space is expected to be rough [32]–[34]. Therefore to explore the area around the reconstructed structures we use an enhanced sampling algorithm, Diffusion Map-directed-Molecular Dynamics (DM-d-MD) [52]. DM-d-MD is an iterative method that uses the ideas of the diffusion map, in particular that the slowest barrier crossing timescale corresponds to the first DC (DC1), to enhance the sampling by increasing the probability that a system will cross free energy barriers. At each iteration, a short swarm of MD trajectories are run from an initial point, a diffusion map calculation is performed on that swarm, and the configuration with the largest DC1 is selected as the ‘frontier point’, which is used as the initial point in the next iteration. DM-d-MD has been illustrated in alanine dipeptide and alanine-12 [52], in which there is a three-orders-of-magnitude speedup of the sampling in comparison to standard MD.

We use this novel combination of techniques to obtain a signaling state ensemble of WT-PYP. This method is unique in that, as far as we can tell, this is the first time results of dynamics with a coarse-grained potential are used to reach a non-native basin, and from coarse-grained structures in that basin all-atom reconstructions are used to initiate a more detailed exploration of the new basin. Our results are in agreement with previous calculations and more recent experimental data. We anticipate the overall strategy presented here to be applied to other systems for which conventional techniques are impractical or impossible.

## Results

### Coarse-grained modeling and LSDMap

We used the DMC method to construct a coarse-grained potential for the ‘activated’ state of PYP, i.e. the state after photo-absorbtion. The details of the construction of the model are given in the Materials and Methods section. Briefly, the DMC model treats each amino acid as a single bead, with non-bonded interactions between beads dependent on the type of two amino acids. To model the photo-activated state of PYP, the nonbonded interactions between the chromophore residue and all others was set to zero, which roughly models the disruption of the hydrogen bonding network within the chromophore binding pocket. A simulation of 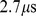 was performed using GROMACS [53], with data collected every 50 ps, and at a temperature sufficient to have many folding/unfolding events.

**Figure 1:**
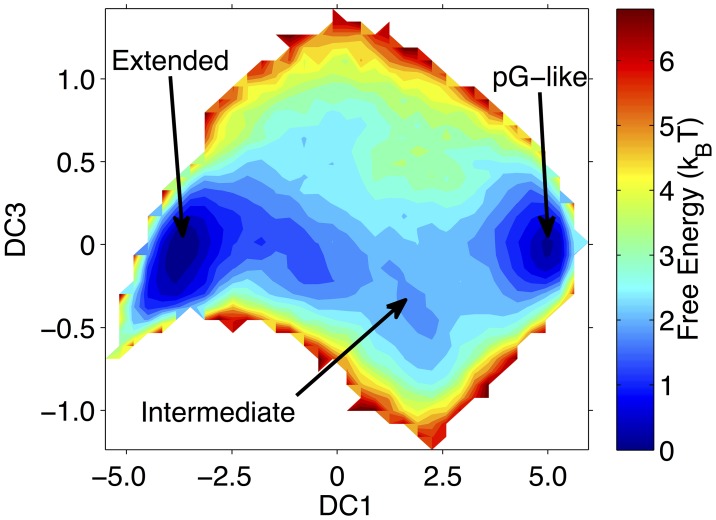
LSDMap of the coarse-grained DMC model of PYP. Structures with large positive first diffusion coordinate (DC1) are globular and similar to the pG state, while structures with large negative DC1 are almost fully extended. The intermediate region near DC1∼2.5 correspond to potential structures for further consideration as candidate signaling pB-state configurations. Figures S1, S2, and S3 in Text S1 show representative configurations from each region. The free energy is shown in terms of DC1 and DC3 to allow for a clearer view of the intermediate region.

In Figure 1 the free energy is shown in terms of the first and third DCs. The slowest collective motion of this DMC system corresponds to a global unfolding of the protein. Structures with a large positive DC1 correspond to configurations very similar to the pG native state, while configurations with a large negative DC1 are unfolded. The figure shows an additional minimum in the intermediate region of the free energy, and configurations within this minimum are good candidate pB-state configurations. Representative structures from each region are presented in Figures S1, S2, and S3 in Text S1. In addition, histograms of the C*α* RMSD to the NMR pG structure are shown in Figure S4 in Text S1. Approximately 1000 coarse-grained structures were collected from the local minimum in free energy near DC1 = 2.5 for further analysis. The free energy is shown in terms of DC1 and DC3 to allow for a clearer view of the intermediate region.

### All-atom reconstruction and DM-d-MD

The DMC model supplied the 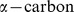 (C*α*) positions for the candidate signaling-state configurations, for which we want to recover the atomic details to more accurately explore the pB region of configuration space. This is accomplished with the Reconstruction Algorithm for Coarse-Grained Structures (RACOGS) [47], which is specially designed to recover all-atom details not only in the native basin, but anywhere in configuration space. One example reconstruction is shown in Figure S5 in Text S1. The ≈1000 reconstructed all-atom configurations are then solvated and equilibrated using previously established protocol [34]. From these, we used the criterion of lowest protein-only potential energy to select 10 structures for further analysis.

**Figure 2:**
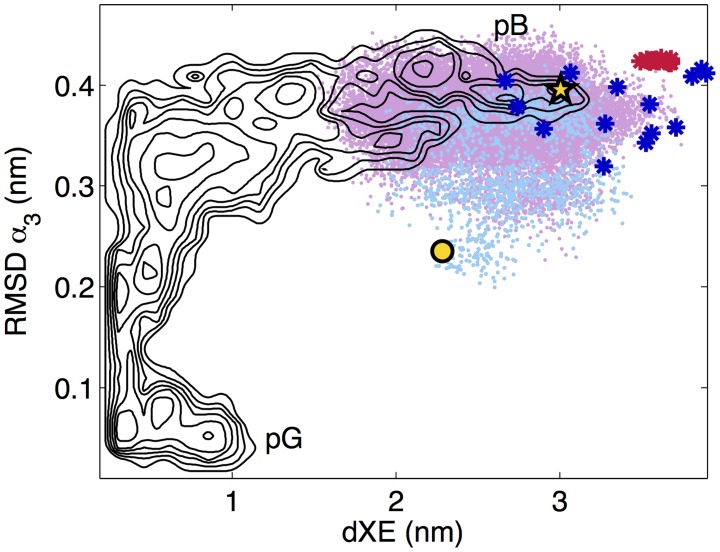
Diffusion Map-directed-Molecular Dynamics (DM-d-MD) and molecular dynamics (MD) results. The initial starting configuration for the DM-d-MD is denoted by the gold circle. The DM-d-MD frontier points are shown in pink, and the minimum-energy frontier point is denoted by a gold star. The MD results from approximately 100 trajectories initiated from the minimum-energy DM-d-MD frontier point are shown in light blue. For reference, the coordinates for the experimental Δ25 (PDB ID 1XFQ) structures are shown as dark blue *, and the experimental WT-PYP (PDB ID: 2KX6) structures as red *. The underlying grey contours are those from Figure 1 of reference [34].

To explore the molecular configuration space around these solvated structures, DM-d-MD [52] is initiated from each equilibrated structure. Previous work has suggested two collective variables: the root mean square deviation (RMSD) of the *α*_3_ helix (residues 41–53) with respect to an ideal helix, and the distance between the GLU46 residue and the pCA chromophore, are good collective variables in which to visualize the system, and that there are a few metastable states in between the pB and pG configurations [34]. These coordinates are used in Figure 2, along with the underlying black free energy contours obtained from previous parallel tempering calculations (See Figure 1 from Vreede, et al. [34]).

In Figure 2 the initial point for the DM-d-MD is shown as a gold circle, the minimum-energy structure from the DM-d-MD as a gold star, and the other DM-d-MD points in light blue. Stand-alone DM-d-MD is an exploratory procedure, and does not yield a Boltzmann distribution of configurations (however techniques such as umbrella sampling can recover the Boltzmann distribution from a set of DM-d-MD frontier points [52]). To recover a local, quasi-equilibrium distribution of the pB signaling state, the lowest energy DM-d-MD frontier point is used to initiate approximately 100 runs of ordinary MD simulations, the results of which are shown in light purple. For the purpose of determining the “lowest-energy” point, the energy was calculated for the protein only, using the Gromos96 43a1 [54] force field. The average length of the runs was 22 ns, and data was collected every 50 ps after the initial 2 ns, yielding a total of 40,209 configurations. This is our pB signaling state ensemble. It should be noted that while the MD results overlap with the metastable minima obtained from previous calculations, as shown in the figure, projection onto a two-dimensional coordinate system can be misleading, and we rely on further analysis below to verify our pB-state ensemble.

The choice of the “lowest energy” DM-d-MD frontier point is simply a convenient choice for the purposes of this study. We show in the Figure S6 in Text S1 that the next few low-energy DM-d-MD points yield similar overall structure by comparing the secondary structure content of various configurations using the Stride algorithm [55]. As discussed below, the secondary structures are all similar to one another, and–with the notable exception of the first 25 residues–similar to the experimental structure. For comparison, the coordinates of the experimental Δ25 and WT-PYP pB-state structures are shown as dark blue and red *, respectively, in Figure 2 The Δ25 structures are much more scattered than the experimental WT-PYP configurations, due to the larger variation in the Δ25 configurations compared to the WT-PYP. Within the 20 structures in the Δ25 set, the relative C*α* RMSD average and standard deviation is 0.41+/−0.07 nm, while for the 14 WT-PYP structures the average and standard deviation is only 0.16+/−0.06 nm. This difference is potentially due to the lack of long-range information in the NMR Δ25 experiments [28].

Interestingly the DM-d-MD explores mostly the upper right-hand region of the RMSD *α*3 – dXE space, and the minimum energy structures are located in the middle of the putative pB region in these coordinates.

## Discussion

### Analysis of pB ensemble

**Figure 3:**
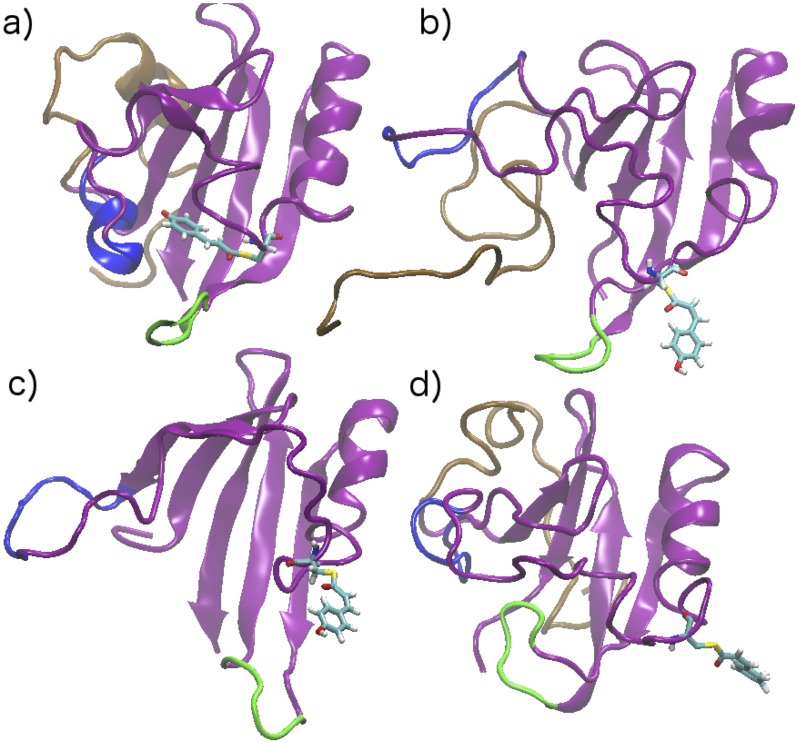
Configuration from a) pG state (PDB ID: 3PHY), b) experimental signaling state of WT-PYP (PDB ID: 2XK6), c) experimental signaling state of Δ25 (PDB ID: 1XFQ), d) representative configuration from our pB ensemble. In all configurations the chromophore (residue 69) is shown explicitly, with the rest of the protein shown as cartoon. The *α*3 helix (residues 43–51) is in blue, the chromophore binding cap (residues 98–103) in green, and the first 25 N-terminal residues, when present, in brown.

Figure 3 displays a configuration in the pG state for reference, a configuration from the experimental WT-PYP pB state, a configuration from the experimental signaling state of Δ25, and a configuration from our pB ensemble. In the pG configuration, the chromophore is tucked inside the chromophore binding pocket, the *α*3 helix (colored in blue) is well formed, and the binding pocket cap (residues 98–103 colored in green) is in place. All three signaling state structures display features known to be associated with the signaling state: the *α*3 helix is unformed and the chromophore is exposed to the solvent. The pB state is in general less well structured than pG configurations, while some secondary structure elements remain intact. Visually, the secondary structure in the Δ25 configuration looks more well-ordered than the experimental WT-PYP and our pB ensemble. In comparing our result with experimental WT-PYP, the amount of retained secondary structure is similar; however the location of the 25 N-terminal residues differs (discussed more below).

To quantify the degree of structural similarity between the pG and pB states, we have computed the relative fluctuations of the 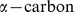 (C*α*) atoms for the various datasets with respect to those of the pG state. Because of the flexible nature of much of the protein in the pB state, this is a better metric than the RMSD between different structures.

These fluctuations are computed by first aligning the corresponding C*α*'s to the last 100 residues of the pG configuration (model 11 of PDB ID: 3PHY [18]), and then calculating the displacement of the C*α*'s from that pG configuration. This was done for the 20 Δ25 structures, the 14 WT-PYP configurations, and the pB ensemble resulting from our method. Only the last 100 residues are used in the alignment and calculation because 1) the first 25 are not present in Δ25 and 2) there is a large difference in the location of these residues in previous calculations and experiment (see below).

**Figure 4:**
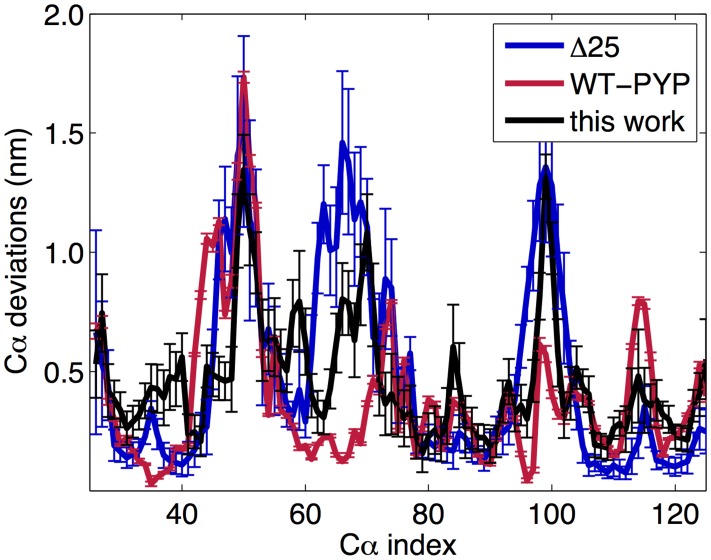
Comparison of the C*α* deviations from the pG configuration for the experimental Δ25 configurations (PDB ID 1XFQ) in black, experimental WT-PYP pB configurations (PDB ID: 2KX6) in green, and our pB ensemble in blue. The horizontal axis is the index number relative to PYP (i.e. the first index of Δ25 is at 25 on the graph). For all three datasets, the regions of the sequence similar to the pG dark state are similar, as are most of the regions where the fluctuations are larger. The primary differences are near the chromophore (residue 69) and are discussed in the main text.

Figure 4 compares the results. For regions of the protein in which the pG secondary structure is preserved in the pB state, for example the *α* helix formed by residues 79–84, the C*α* displacement is minimal. However in regions of the protein where structure is lost, for example the *α*3 helix in residues 79–84, the fluctuations are larger. There is general agreement among all three datasets. The two main conserved regions are the helix in residues 76–85, and the central *β* sheet, and can be seen in all three signaling-state structures in Figure 3.

From Figure 4, near the chromophore region the experimental WT-PYP configurations are more similar to the pG state, the Δ25 configurations fluctuate most, and our configurations are in between. In the experimental WT-PYP configurations, the loop containing the chromophore only moves enough to allow the chromophore to be flipped out of the binding pocket, while in the Δ25 configurations the structure is comparatively more extended, leading to larger deviations from the pG dark state.

The most significant difference between the WT-PYP pB state and our ensemble is the location of the first 25 N-terminal residues. The experimental configuration shows an open binding pocket, and the N-terminal residues across the pocket [28]. Our configurations, as well as previous calculations [32], have the N-terminal residues behind the central *β* sheet, and away from the binding pocket. This difference is most likely due to the force-field used in simulation. Due to hydrophobicity, it is unlikely that a molecular dynamics simulation will explore open configurations such as that shown in panel b of Figure 3. Indeed we have solvated and equilibrated one of the configurations from PDB ID 2KX6 (one of the red *s in Figure 2) and performed 20 independent 30-ns simulations. In most of the simulations, the extended N-terminal tail moves toward and into the open chromophore binding pocket. Representative configurations are shown in Figure S8 in Text S1. Figure S7 in Text S1 shows the results of a Stride [55] secondary structure calculation at various snapshots both during one of these simulations and during a simulation from our signaling ensemble.

The location and interaction of the first 25 N-terminal residues with the rest of the protein is still an open question for this system. These interactions are important for understanding WT-PYP, and the different kinetics in the Δ25 system, which has a pB state lifetime roughly 100 times longer than that of WT-PYP [22].

### N-terminus, *α*3 helix, binding pocket cap, and the central *β* sheet

Obviously the difference in Δ25 and WT-PYP kinetics is due to the absence of the first 25 N-terminal residues in Δ25. Experimental results on Δ25 show that even in the pG state the *α*3 helix is unstable compared to the pG state in WT-PYP [27]. In addition, Δ25 even exhibits unfolding in biological conditions without any photo activation [22], which is not observed for WT-PYP.

Previous calculations show reformation of the *α*3 helix is a bottleneck in recovery of the pG state from the pB, and have suggested that the chromophore cannot form the needed contacts in the binding pocket if this helix is not well formed [33]. One possibility is that the N-terminal residues in WT-PYP facilitate the formation of this helix, increasing the recovery rate of pG relative to Δ25. Our pB ensemble shows interactions between the N-terminal residues and the *α*3 helix in the form of hydrogen bonds. 70% of the configurations have hydrogen bonds between ASN43 and GLN22, 59% have hydrogen bonds between ALA44 and ASP24, and 54% have hydrogen bonds between ALA44 and GLY25. It is possible that these interaction slow the reformation of the helix, speeding the recovery of pG in WT-PYP.

In the pG state, the first 25 residues are separated from the chromophore binding pocket by the central *β* sheet (see Figure 3). It is proposed that interactions through this *β* sheet lead to a stabilization of the chromophore binding pocket. In our simulations (and in those reported previously [32]), the N-terminus in the pB ensemble is in van der Waals contact with the back side of the central *β* sheet. Such contact may encourage the reformation of the chromophore binding pocket, increasing the pG recovery rate in PYP.

There is a possibility of allosteric interactions that drive the transition to and from the signaling state. To begin to investigate this issue, we have analyzed our signaling state ensemble using a generalized correlation [56], [57]. The results of this analysis are shown in the Figures S9 and S10 in Text S1, and suggest a potential bridging interaction of the chromophore binding cap with the *α*3 helix and the chromophore region. This is a direction we plan to pursue in future work.

As pointed out above, the location of the N-terminal residues differs between experiment and theory. At this point it is unknown if these differences are due to the force fields used in calculation, differences in the experimental sample preparation details compared to those of the calculation (the specifics of the PYP photo cycle are known to be environmentally dependent [1]), or to something else entirely. More work, both experimental and computational, is required to fully understand these aspects of the WT-PYP and Δ25 photo cycles.

### Conclusions

We have presented a novel combination of techniques: DMC coarse-grained modeling, LSDMap, RACOGS all-atom reconstruction, and DM-d-MD to obtain an ensemble of signaling state structures for WT-PYP. This amalgam of methods allows for an initial bird's-eye view of the free energy landscape, followed by a “zooming-in” to a region of interest. Such a process is ideal for the PYP system for several reasons. The timescales present are too long for conventional MD, and even advanced sampling methods such as parallel tempering [32] and transition path sampling [34] methods are pushed to their limits, making coarse-grained methods useful. The coarse-grained method we employ includes the effect of non-native interactions, which are thought to be important here, and only requires the structure of the pG dark state as an input. The LSDMap analysis method extracts collective coordinates directly from the results of the coarse-grained simulation with which to analyze the free energy landscape, without the need to rely on calculation [34] or chemical intuition to arrive at collective coordinates in which to analyze the free energy. Once coarse-grained structures are found in a region of interest in the LSDMap free energy, the RACOGS method is used to recover all-atom structural details. This method is designed to work well not only near native-like configurations, but to provide physically realistic structures anywhere in the landscape. Finally DM-d-MD allows for a rapid exploration of the newly located region of configuration space. This procedure should prove useful in other systems with long timescales and unknown free energy minima.

Our results compare favorably with previously reported experimental and computational findings, which serves as a validation of our techniques and support for previous results. However uncertainties remain concerning the PYP photo cycle, in particular the role of the first 25 N-terminal residues. There is agreement between the current and previous computational results [32]; however both sets of computational results differ from experiment in the structure of the fist 25 N-terminal residues [9], [28]. More work is needed to understand this aspect of the PYP photo cycle.

## Materials and Methods

### DMC coarse-grained model

We use a coarse-grained modeling method [38], termed the DMC method, to model the PYP protein in its “activated” state, i.e. after photon absorption and isomerization of the chromophore. The method itself is generally applicable to any system for which a native structure is available, and accounts for (in an approximate fashion) both the geometrical differences between various residue types and minimizes the energetic frustration of the folded structure.

The DMC method treats each residue as a single bead, with the potential energy written as
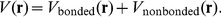
(1)


The local interactions comprise bonds, angles, and dihedral terms,
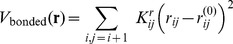
(2)

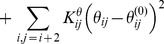
(3)

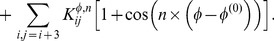
(4)


The nonbonded terms
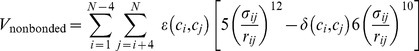
(5)are chosen as follows. The distances 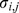 are determined both by the type of residue-residue interaction, i.e. their color 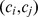, and their relative distance along the chain. The value of 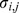 for a particular pair of residues is extracted from a probability distribution 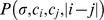. For each possible interaction 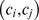, three different distance histograms are constructed: one for 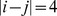, one for 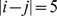, and one for 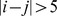, based on the frequency of the C*α* - C*α* distances between residue pair types in native structures in the PDB. The value of 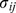 is then taken from the appropriate histogram for the two residues in question.

The well depth for each pair of residues 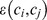, and a factor that determines if the interaction is attractive or purely repulsive, 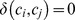 or 1, is different for each type of interaction, independent of their relative distance along the chain. These values are determined through an iterative procedure using Monte Carlo simulated annealing and perceptron learning [58] to maximize the energy gap between the native structure of the protein and similar globular misfolded states [35], [59], [60]. See Das, et al. [38] for further details on the general algorithm for the DMC coarse-grained modeling technique.

The procedure outlined above was used here, with the crystal structure of the pG state as the native structure. The result is a DMC model for the dark state. In order to arrive at a model for the photo-activated state, i.e. after photon absorption, the nonbonded interactions between the chromophore residue (CYS69) and all of the other residues were set to zero, i.e. 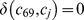, 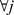. Turning off these interactions approximates the known behavior of photo-activated WT-PYP, namely that the hydrogen bonding network around the chromophore is disrupted as a consequence of photo-activation.

### LSDMap

We use the LSDMap [43] technique to understand the free energy landscape mapped out during the DMC coarse-grained simulation. LSDMap is a kernel-based method of approximating numerical solutions to the Fokker-Planck equation. The method takes as input a set of molecular coordinate trajectories and outputs a set of collective coordinates, termed diffusion coordinates, which are ordered in terms of their relative timescales: the first diffusion coordinate corresponds to the barrier crossing with the longest rate, the second diffusion coordinate is the second longest, etc. The LSDMap code is available via SourceForge [61].

### RACOGS

We use the reconstruction algorithm for coarse-grained structures (RACOGS) [47] in order to recover the all-atom configuration of candidate signaling structures from the DMC coarse-grained model. This method was developed to provide physically realistic reconstructions in any region of configuration space (i.e. not only near or within the native-state basin). The technique involves several steps: 1) backbone reconstruction, 2) side chain placement, 3) side chain minimization, 4) addition of hydrogens, and 5) all-atom minimization. This minimization in step 3 detects and performs energy minimization directly on high-energy side chains. This has the effect of reducing any bias that might be present from using a rotamer library taken from native or near-native configurations, therefore making the method more likely to produce realistic structures outside of the native basin. See reference [47] for further details.

### MD solvation and equilibration

The vacuum structures resulting from the RACOGS algorithm are solvated with water and Na+ counter ions in GROMACS [53], using the same force field (Gromos96 43a1 [54], Simple Point Charge (SPC) water model [62]), and general topology as previous work [32]. The system is equilibrated as in the initial preparation of reference [34]. Of the approximately 1000 structures reconstructed, the lowest energy configurations after equilibration were used to initiate DM-d-MD calculations described below.

### DM-d-MD

We use the recently proposed diffusion map-directed-molecular dynamics (DM-d-MD) [52] procedure to explore the region of configuration space around the reconstructed all-atom configurations. DM-d-MD is an iterative enhanced sampling method in which a swarm of short molecular dynamics simulations are performed at each iteration, a diffusion map calculation is performed on the resulting trajectories, and the furthest point from the swarm is determined from the first diffusion coordinate. This furthest point (the “frontier” point) is then used to initialize the swarm for the next iteration. By restarting the next iteration from the frontier point, the technique significantly increases the likelihood that the system will escape from local free energy minima.

## Supporting Information

Text S1 Includes additional figures on: the coarse grained structures from different regions of the PYP folding landscape; a comparison of the similarity of the the different regions of the free energy of the coarse-grained model; example of a reconstructed configuration; secondary structure analysis for different configurations; the results of molecular dynamics performed from the pB structure for the experimental WT-PYP; and the results on the generalized correlation analysis on the predicted pB ensemble.
(PDF)
