# Correction: Multiscale Approach to the Determination of the Photoactive Yellow Protein Signaling State Ensemble

**DOI:** 10.1371/journal.pcbi.1005770

**Published:** 2017-09-25

**Authors:** The PLOS Computational Biology Staff

Dr. Wouter Hoff should be included as a co-author of this paper.

The author’s affiliation is as follows:

Department of Microbiology and Molecular Genetics, Oklahoma State University, Stillwater, Oklahoma, United States of America

The author list should read as follows:

Mary A. Rohrdanz, Wenwei Zheng, Wouter Hoff, Bradley Lambeth, Jocelyne Vreede, Cecilia Clementi

The Author Contributions section should read as follows:

Conceived and designed the experiments: MAR WZ CC WH. Performed the experiments: MAR WZ BL. Analyzed the data: MAR WZ CC. Contributed reagents/materials/analysis tools: JV. Wrote the paper: MAR CC.

The funding statement should read as follows:

This work was funded by the Welch Foundation C-1570, NSF CHE-1152344, NSF CHE-1265929, NSF OCI-1053575, NSF EIA-0216467, NIH NCRR S10RR02950. WDH is supported by NSF grants MCB-1051590, MRI-1338097, and CHE-1412500. The funders had no role in study design, data collection and analysis, decision to publish, or preparation of the manuscript.

The authors confirm that the addition of this co-author does not affect the competing interest statement.

## References

[1] A. Rohrdanz M, Zheng W, Lambeth B, Vreede J, Clementi C (2014) Multiscale Approach to the Determination of the Photoactive Yellow Protein Signaling State Ensemble. PLoS Comput Biol 10(10): e1003797 doi:10.1371/journal.pcbi.1003797 25356903 10.1371/journal.pcbi.1003797 PMC4214557

