# Supplementary material for: Multiscale Approach to the Determination of the Photoactive Yellow Protein Signaling State Ensemble
Source: PLoS Comput Biol. 2014 Oct 30;10(10):e1003797. doi: 10.1371/journal.pcbi.1003797 (PMC4214557; doi:10.1371/journal.pcbi.1003797)
Supplement: Text S1 — Includes additional figures on: the coarse grained structures from different regions of the PYP folding landscape; a comparison of the similarity of the the different regions of the free energy of the coarse-grained model; example of a reconstructed configuration; secondary structure analysis for different configurations; the results of molecular dynamics performed from the pB structure for the experimental WT-PYP; and the results on the generalized correlation analysis on the predicted pB ensemble. (PDF) [file pcbi.1003797.s001.pdf]

Supporting Information

Multiscale approach to the determination of the photoactive yellow protein signaling state ensemble

Rohrdanz, Zheng, Lambeth, Vreede, and Clementi

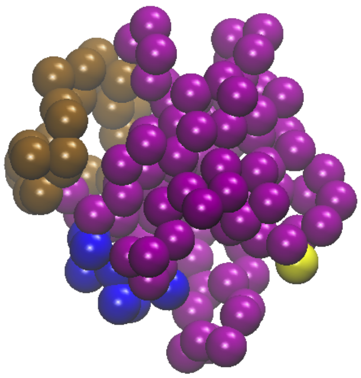

S 1: Example configuration from the pG-like region of Figure 1 in the main text. The first 25 residues are colored in brown, the α_3 helix in blue, and the chromophore residue in yellow.

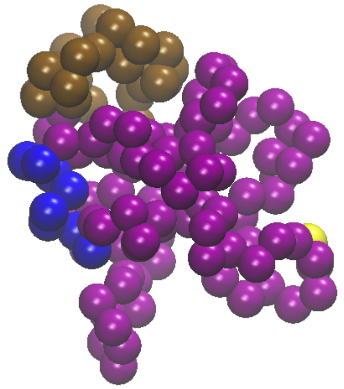

S 2: Example configuration from the intermediate region of Figure 1 in the main text. The first 25 residues are colored in brown, the α_3 helix in blue, and the chromophore residue in yellow.

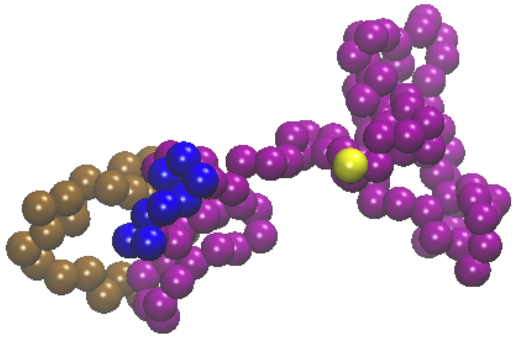

S 3: Example configuration from the extended region of Figure 1 in the main text. The first 25 residues are colored in brown, the α_3 helix in blue, and the chromophore residue in yellow.

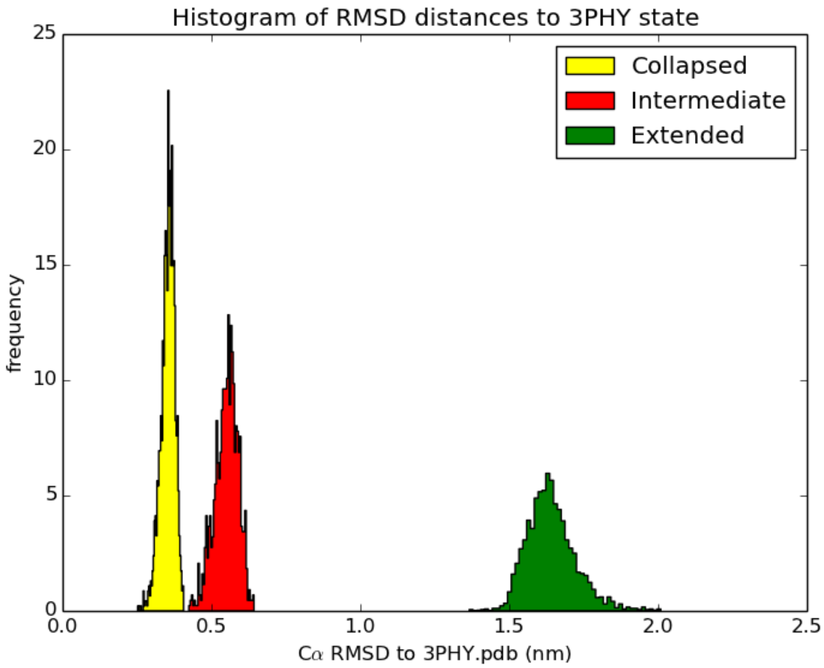

S 4: In order to compare the similarity of the different regions of the free energy of the DMC model shown in Figure 1 of the main text, we calculated the Cα RMSD of the configurations in each of the three regions with respect to the NMR configuration of the dark state, PDB ID: 3PHY. Histograms of the results for each of the three regions are shown. The pG-like region (yellow) is closest to the 3PHY configurations, with differences attributable to the different interactions between the chromophore residue in our photo-activated DMC model and the rest of the protein. The intermediate region (red) is less similar, and the extended region (green) is quite different, as expected.

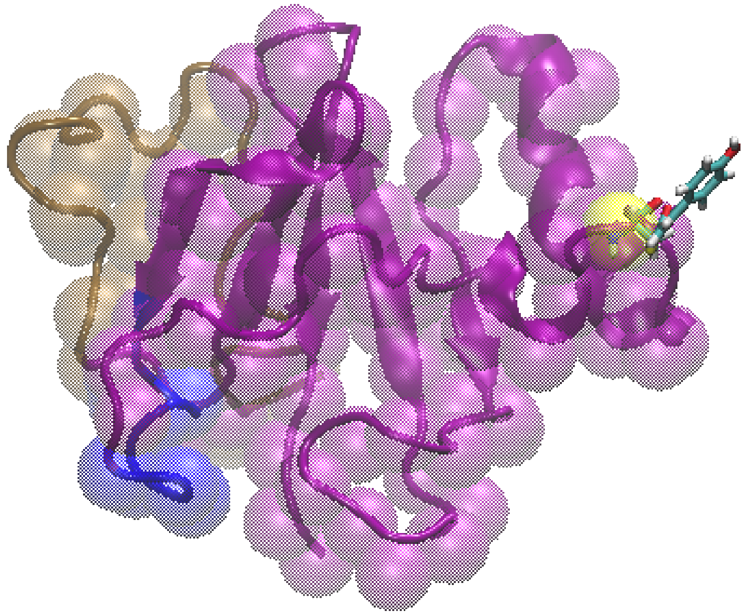

S 5: Overlay of typical DMC model configuration and its corresponding all-atom reconstruction. The first 25 residues are colored in brown, the α_3 helix in blue, and the chromophore residue in yellow. In the all-atom reconstruction, the protein is shown in the 'New Ribbon' representation, and the chromophore residue shown as 'Licorice'.

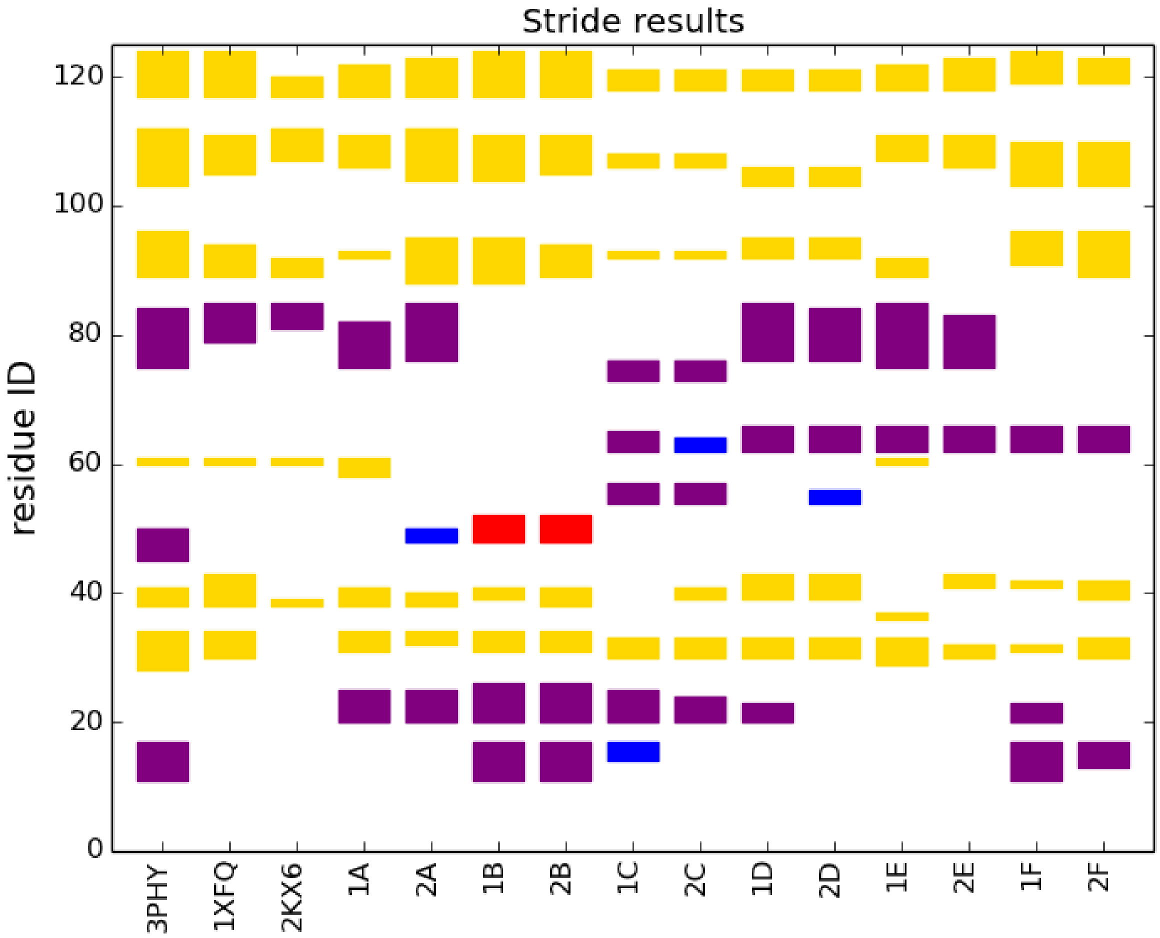

S 6: Secondary structure as calculated by the stride algorithm [1] for various configurations. Purple bars correspond to α helices; yellow bars correspond to β strands; red bars correspond to π helices; blue bars correspond to 3-10 helices. 3PHY is the dark state of WT-PYP [2]. 1XFQ is the photo-activated state of the $\Delta 25$ mutant [3]; the residue numbering has been shifted to coincide with WT-PYP. 2KX6 is the photo-activated state of WT-PYP [4]. The remaining columns are the secondary structure for the 2 lowest-potential energy configurations from 6 DMDMD runs. There is general agreement between the DM-d-MD points and the 2KX6 experimental structure, with the notable exception of the first 25 residues.

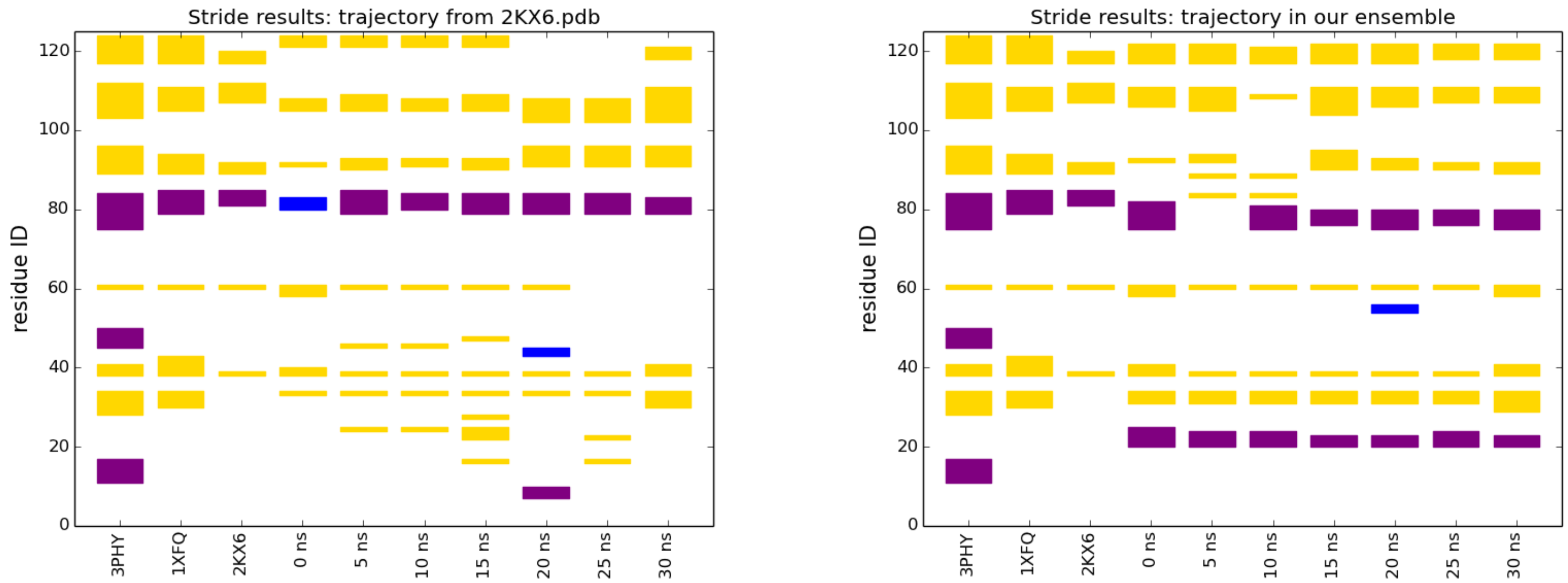

(a) (b)

S 7: Comparison of secondary structure as calculated by the stride algorithm [1] for a trajectory initiated from 2KX6.pdb (Left) and a trajectory from one of the configurations is our signaling state ensemble (Right). In both panels, the vertical axis is the residue number, purple bars represent α helices, yellow bars represent β strands, and blue bars represent 3-10 helices. The first column is the calculation for 3PHY.pdb (the dark-state experimental result); the second column is for 1XFQ.pdb (the $\Delta 25$ mutant, numbered to alighn with WT-PYP; the third column is for 2KX6.pdb (the experimental signaling-state result). The remaining columns are from simulation: left for a simulation initiated from 2KX6, right for a simulation that is part of our signaling state ensemble. There is a good correspondence between the two simulations, with the exception of the 25 N-terminal residues. The configurations for the 2KX6 trajectory at times 0 ns and 30 ns are shown in figure S8

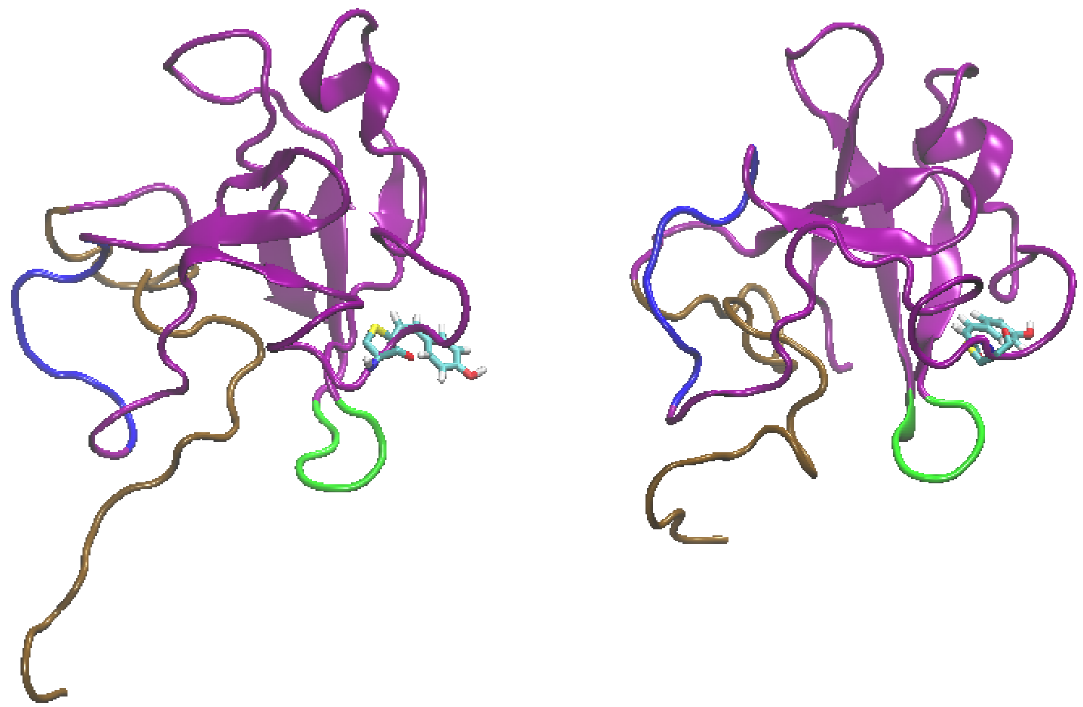

S 8: Configurations from a representative simulation initiated from the experimental 2KX6.pdb configuration. Left: 0 ns, i.e. after solvation and equilibration of the PDB structure. Right: configuration after 30 ns of simulation. The extended first 25 N-terminus residues, which are lying across the chromophore binding pocket and extending outward from the rest of the configuration at t = 0, move up and into the open binding pocket. The first 25 residues are colored brown, the $\alpha3$ helix in blue, and the binding pocket cap in green. The chromophore is shown in licorice.

1 Generalized Correlation

The degree of allosteric interaction that may be present in the PYP system is still an open question. To begin to address this issue, we have used the generalized correlation method of Lange and Grubmüller [5], that estimated the mutual information as in Kraskov, et al. [6]. We present results for the first algorithm in figure S9; the other algorithm looks similar.

The results for our signaling state ensemble are shown in figure S9. For the most part, areas of high correlation correspond to regions near to one another in the overall structure, e.g. adjacent β strands. However, there is a "bridging" region of correlation highlighted by the yellow ellipse in Figure S9. Here there is correlation between residues 46–54 (roughly the $\alpha 3$ helix) and residues 95–103 (roughly the chromophore binding pocket cap). There is also correlation between the binding pocket cap residues and residues 64–71, which are surrounding the chromophore. This network of correlations is interesting because previous computational work from Vreede, et al. [7] has suggested that the reformation of the $\alpha 3$ helix is key for the recovery of the dark state from the signaling state.

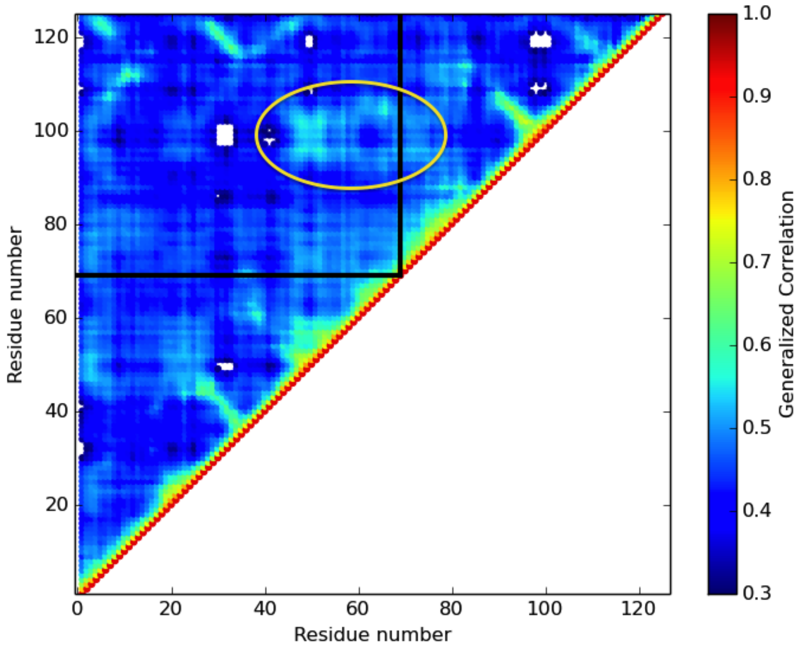

S 9: The degree of correlation in a set of 50 9-ns simulations determined using Lange and Grubmüller [5] and Kraskov, et al. [6]. The bridging interaction is highlighted by the yellow ellipse. Here regions near the the $\alpha 3$ helix (roughly residues 46-54) are correlated with residues 95-103, which correspond to the region near the chromophore binding pocket cap. This binding pocket cap is correlated with residues 64-71, which are near the chromophore (residue 69). See also Figure S10

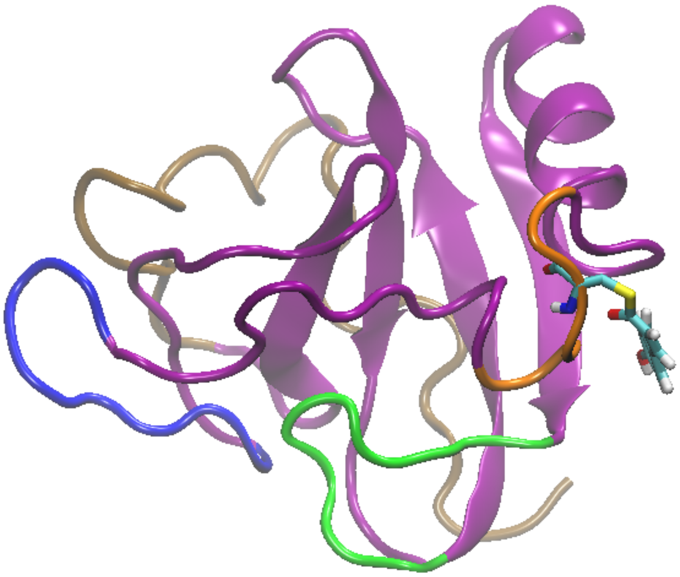

S 10: Example signaling-state structure highlighting the regions involved in the bridging correlation shown in Figure S9. Based on the generalized correlation shown in Figure S9, the $\alpha 3$ helix, approximately residues 46–54 (blue), is correlated with residues 95–103 (green), which roughly correspond to the chromophore binding pocket cap. The binding cap residues are correlated with residues 64–71 (orange), which contain the chromophore.

References

[1] Yang Shen, M Heinig, Ad Bax, and D Frishman. STRIDE: a web server for secondary structure assignment from known atomic coordinates of proteins. *Nucleic Acids Res.*, 32(Web Server):W500–W502, July 2004.

[2] Petra Düx, Gilles Rubinstenn, Geerten W Vuister, R Boelens, Frans A A Mulder, Karl Hård, Wouter D Hoff, Arthur R Kroon, Wim Crielaard, Klaas J Hellingwerf, and Robert Kaptein. Solution Structure and Backbone Dynamics of the Photoactive Yellow Protein ,. *Biochemistry*, 37(37):12689–12699, September 1998.

[3] C Bernard, K Houben, N Derix, D Marks, M Vanderhorst, Klaas J Hellingwerf, R Boelens, Robert Kaptein, and N Vannuland. The Solution Structure of a Transient Photoreceptor Intermediate: Δ25 Photoactive Yellow Protein. *Structure*, 13(7):953–962, July 2005.

[4] Pradeep L Ramachandran, Janet E Lovett, Patrick J Carl, Marco Cammarata, Jae Hyuk Lee, Yang Ouk Jung, Hyotcherl Ihee, Christiane R Timmel, and Jasper J van Thor. The Short-Lived Signaling State of the Photoactive Yellow Protein Photoreceptor Revealed by Combined Structural Probes. *J Am Chem Soc*, 133(24):9395–9404, June 2011.

[5] Oliver F Lange and Helmut Grubmüller. Generalized correlation for biomolecular dynamics. *Proteins*, 62(4):1053–1061, December 2005.

[6] Alexander Kraskov, Harald Stögbauer, and Peter Grassberger. Estimating mutual information. *Phys Rev E*, 69(6):066138, June 2004.

[7] Jocelyne Vreede, Klaas J Hellingwerf, and Peter G Bolhuis. Helix formation is a dynamical bottleneck in the recovery reaction of Photoactive Yellow Protein. *Proteins*, 72(1):136–149, July 2008.
